# Metagenomics reveals impact of geography and acute diarrheal disease on the Central Indian human gut microbiome

**DOI:** 10.1080/19490976.2020.1752605

**Published:** 2020-05-27

**Authors:** Tanya M. Monaghan, Tim J. Sloan, Stephen R. Stockdale, Adam M. Blanchard, Richard D. Emes, Mark Wilcox, Rima Biswas, Rupam Nashine, Sonali Manke, Jinal Gandhi, Pratishtha Jain, Shrejal Bhotmange, Shrikant Ambalkar, Ashish Satav, Lorraine A. Draper, Colin Hill, Rajpal Singh Kashyap

**Affiliations:** a NIHR Nottingham Biomedical Research Centre, Nottingham University Hospitals NHS Trust and the University of Nottingham, Nottingham, UK; b Nottingham Digestive Diseases Centre, School of Medicine, University of Nottingham, Nottingham, UK; c School of Life Sciences, University of Nottingham, Nottingham, UK; d APC Microbiome Ireland, University College Cork, Cork, Ireland; e School of Veterinary Medicine and Science, Sutton Bonington Campus, University of Nottingham, Leicestershire, UK; f Advanced Data Analysis Centre, Sutton Bonington Campus, University of Nottingham, Leicestershire, UK; g Leeds Teaching Hospitals NHS Trust and University of Leeds, UK; h Biochemistry Research Centre, Central India Institute of Medical Sciences, Nagpur, India; i Department of Clinical Microbiology and Infection, King’s Mill Hospital, Sherwood Forest Hospitals NHS Trust, Sutton in Ashfield, UK; j Mahatma Gandhi Tribal Hospital, Dharni, India

**Keywords:** Gut microbiome, antibiotic resistome, virome, diarrhea, *Clostridioides difficile*, Central India

## Abstract

**Background:**

The Central Indian gut microbiome remains grossly understudied. Herein, we sought to investigate the burden of antimicrobial resistance and diarrheal diseases, particularly *Clostridioides difficile*, in rural-agricultural and urban populations in Central India, where there is widespread unregulated antibiotic use. We utilized shotgun metagenomics to comprehensively characterize the bacterial and viral fractions of the gut microbiome and their encoded functions in 105 participants.

**Results:**

We observed distinct rural-urban differences in bacterial and viral populations, with geography exhibiting a greater influence than diarrheal status. *Clostridioides difficile* disease was more commonly observed in urban subjects, and their microbiomes were enriched in metabolic pathways relating to the metabolism of industrial compounds and genes encoding resistance to 3^rd^ generation cephalosporins and carbapenems. By linking phages present in the microbiome to their bacterial hosts through CRISPR spacers, phage variation could be directly related to shifts in bacterial populations, with the auxiliary metabolic potential of rural-associated phages enriched for carbon and amino acid energy metabolism.

**Conclusions:**

We report distinct differences in antimicrobial resistance gene profiles, enrichment of metabolic pathways and phage composition between rural and urban populations, as well as a higher burden of *Clostridioides difficile* disease in the urban population. Our results reveal that geography is the key driver of variation in urban and rural Indian microbiomes, with acute diarrheal disease, including *C. difficile* disease exerting a lesser impact. Future studies will be required to understand the potential role of dietary, cultural, and genetic factors in contributing to microbiome differences between rural and urban populations.

## Introduction

The human gut houses a complex microbial ecosystem referred to as the microbiome, which includes prokaryotic, eukaryotic, and viral components. While the bacterial components of the microbiome have received considerable attention, comparatively little is known about the composition and physiological significance of human gut-associated bacteriophage populations, otherwise known as the phageome.^1^ Moreover, despite the growing global burden of antibiotic resistance to modern health care, very few studies have directly^2,3^ or indirectly (through analyzing urban sewage)^4^ examined the antibiotic resistomes of human fecal metagenomes. Such paucity of data prevents a complete understanding of the global burden and transmission of antimicrobial resistance (AMR), which is essential to support national and global priority setting, public health actions, and treatment decisions. Although recent years have seen an explosion of gut microbiome studies in rural pre-industrialized societies such as hunter-gatherer and other geographically diverse populations,^5–10^ little is known about microbial variability and its implications for health and disease in other underrepresented populations in South America, Africa, and regions in Asia, particularly India, where there is a scarcity of microbiome data in diarrheal and other populations.^11–14^ Diarrheal diseases are a major cause of morbidity and mortality in India, making identification of etiological agents of utmost importance.^15–18^

In India, there is tremendous opportunity to study highly diverse communities with varied geographic distribution, dietary habits, and socioeconomic stratification. Some of these communities, including a large tribal population, remain dependent on hunting, agriculture and fishing with their own culture, tradition, dietary habits, language, and genetic make-up. Recently, studies have begun to explore the Indian gut microbiome including that of the country’s scheduled tribes, principally using 16S rRNA gene amplicon sequencing methods to profile mainly gut bacterial diversity in rural and urban healthy populations^11-14^ with only a few reports employing whole-genome shotgun metagenomic sequencing approaches.^19,20^ Whilst the majority of the aforementioned studies have analyzed small population cohorts from Northern, Southern, and Western Indian territories, there is a dearth of information characterizing the gut microbiomes of Central Indian populations. Furthermore, little is known about the burden of *Clostridioides difficile* infection (CDI) in India, the leading worldwide cause of antibiotic-associated diarrhea in hospitalized and community populations^21-25^ and its impact on Indian metagenomes. Profligate, unregulated antibiotic use and inappropriate prescribing suggest that CDI could be widespread in India, the world’s largest consumer of antibiotics.^26^

Via a preexisting research partnership between the University of Nottingham and the Central India Institute of Medical Sciences (CIIMS), we were able to define the gut bacteriome, antibiotic resistome, and virome in understudied rural and urban diarrheal and control populations in Central India. CIIMS has established multisite links with several hospital laboratories in the surrounding district of Nagpur, as well as a satellite laboratory in the Mahatma Gandhi Tribal hospital, Melghat, home to the Korku tribe of agriculturalists. We also concentrated on the pathogen *Clostridiodies difficile* and assessed its impact on the gut microbiome.

Our results indicate that the rural habitants of Melghat show a *Prevotella*-dominant microbiome compared with the urban population of Nagpur, which is enriched with *Bacteroides spp*. Urbanization is associated with functional enrichment of genes involved in xenobiotic and lipid metabolism. Although a core set of AMR genes are detectable in the Korku population, Nagpurian urbanites display a much higher burden of AMR overall. Viral diversity and composition are more influenced by geography than diarrheal status, with urban- and rural-specific phage populations linked to bacterial hosts through CRISPR spacer identification. *C. difficile* is principally detected in the urban and peri-urban exposed antibiotic populations, many of which carry AMR genes to virtually every class of antibiotic.

## Results

### Cohort Characteristics

For our fecal metagenome study in which we were comparing urban vs rural microbiome profiles and assessing impact of diarrhea and CDI, we analyzed fecal samples collected from 105 Central Indian participants comprising 35 rural (12 with diarrhea) and 70 urban (46 with diarrhea) participants from Melghat and Nagpur districts, respectively (Supplementary Table 1 and Supplementary metadata). We selected an enriched set of fecal DNA samples derived from diarrheal samples that had previously tested positive in diagnostic *C. difficile* immunoassays for whole-genome shotgun sequencing (WGS). Of these diarrheal samples, 63% (29/46; urban) and 25% (3/12; rural) had tested positive for toxigenic *C. difficile* in the C. DIFF QUIK CHEK assay.

**Figure 1.**
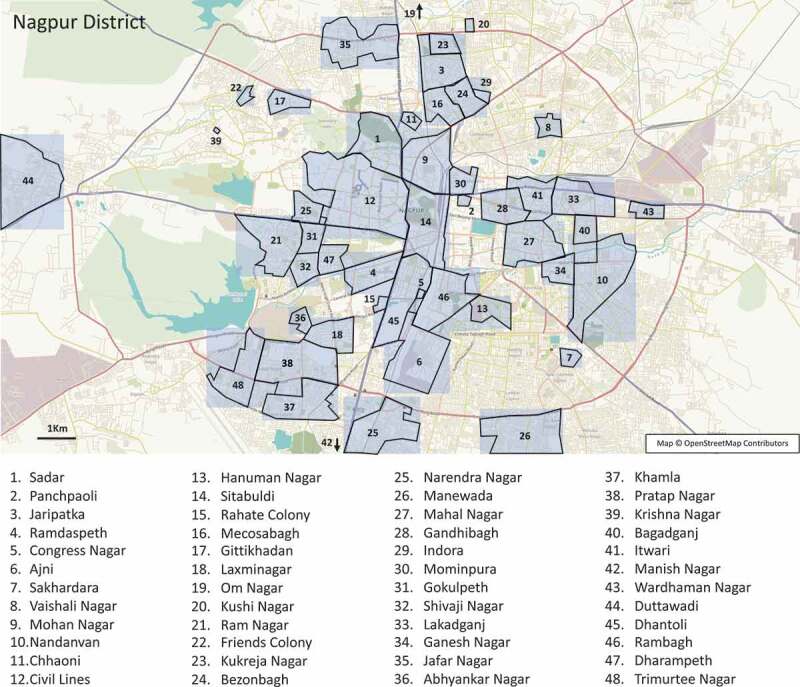
Nagpur District. Mapped locations of study participant home residences in Nagpur district.

Stool samples received centrally by CIIMS were collected at recruitment over 13 months from the 1^st^ of March 2017 to 30^th^ April 2018 from participants resident at 48 sites in Nagpur district (Figure 1) and 19 participating rural villages in Melghat (Supplementary Figure 1), 3 of which were very small villages and are not marked on Google maps. The mean duration of diarrhea for urban diarrheal group (n = 34) was 5.2 days (SD 2.7 days). The mean age of participants was greater for urban (42 years) versus rural (35.6 years) participants, *p* = .01, with a lower percentage of females represented in the urban and rural control groups compared to the diarrheal groups which did not reach statistical significance. Mean body mass index (BMI) [weight (kg)/height (m) squared] was also higher in the urban (21.8) compared with rural (19.3) participants group, *p* < .0001). It was noteworthy that one-third of participants in the urban non-diarrheal control group had received antibiotics in the 3 months prior to recruitment, although none were taking antibiotics when sampled. The vast majority of participant housing in the rural areas was deemed to be of poor quality based on a lack of piped water supply (water tank only), no access to latrines, limited electricity supply (<18 hours/day) and small living space (Supplementary Figure 2), whereas just over half of the urban cohort resided within housing of good quality, as reflected in access to Corporation tap water, longer duration electricity supply (>18 hours/day) and larger living quarters. A higher proportion of rural participants kept domestic animals within their living quarters (cattle, goats, chickens) compared with their urban counterparts.

Overall, significant confounding associations were observed between geographic location and several other study variables. Consequently, we focussed our analyzes primarily on geographic location, with the understanding this accounts for both subject specific and environmental factors.

### Rural subjects have a distinct microbiome when compared with urban subjects

**Figure 2.**
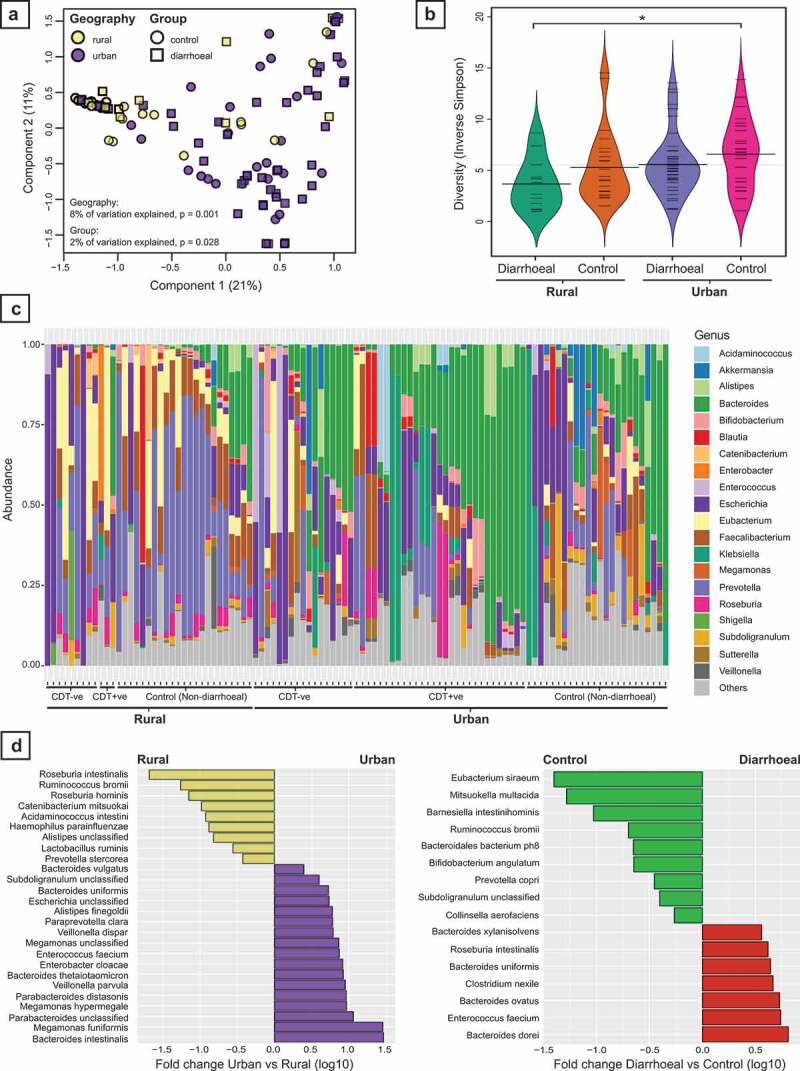
Variations in the gut microbiota by geographic location and diarrheal status. (A) Principal coordinates analysis (PCoA) of microbiota profiles based on Bray–Curtis Dissimilarity of species-level taxonomic abundance. Subject profiles vary by both geographic location and diarrheal status. (B) Comparison of microbial diversity between diarrheal and non-diarrheal control subjects from both rural and urban geographic locations. * p.corr = 0.05. (C) Summary of genus-level taxonomic profiles by subject. Subjects are grouped by geographic location and diarrheal status, with diarrheal subjects further subdivided into *C. difficile* toxin positive (CDT +ve) and negative (CDT – ve). Bacteroides dominant profiles are more frequent in urban subjects, while Prevotella dominant profiles are more frequent in rural subjects. (D) Differentially abundant taxa at species-level based on either geographic location (left, rural vs urban control subjects) or diarrheal status (right, non-diarrheal controls vs diarrheal). All taxa shown are significantly different between groups based on generalized linear models with FDR corrected *p* < .05.

Principal coordinates analysis was performed on a Bray–Curtis Dissimilarity matrix of the species-level taxonomic profiles (n = 105), excluding viral taxa. Urban (n = 70) and rural (n = 35) subjects separated well along with the 1^st^ principal component (Figure 2A) but diarrheal status (control n = 47 vs. diarrheal n = 58) did not appear to have as much influence on sample clustering. This observation was confirmed by PERMANOVA which indicated that geographic location (urban vs rural) accounted for 7.7% of the variation between samples (F = 8.67, *p* = .001) while diarrheal status accounted for a further 1.7% (F = 1.94, *p* = .028). Including *C. difficile* toxin status and recent antibiotic exposure in the model accounted for an additional 2.1% (F = 2.48, *p* = .005) and 1.4% (F = 1.62, *p* = .09) of variation, respectively. Considering other demographic variables of interest, including age, gender, BMI, housing quality, and animal ownership when combined with geography, only age (2.1%, F = 2.41, *p* = .008) contributed significantly to the residual variation explained, reflecting the strong association of these variables with study location.

Sample alpha diversity was calculated using the Inverse Simpson Index for the taxonomic abundances at species level and compared between control and diarrheal subjects from either an urban or rural location (Figure 2B). Rural diarrheal subjects had the lowest diversity (n = 12, mean 3.66 ± 2.5) which was significantly lower than urban control subjects who had the highest diversity (n = 24, 6.75 ± 3.5, p.corr = 0.05).

Individual taxonomic profiles showed a high level of heterogeneity at genus level both within and between study groups (Figure 2C). Overall, profiles from urban areas tended to be dominated by *Bacteroides spp*. with 25/70 urban subjects having a relative abundance of greater than 30% compared to only 3/35 rural subjects (Chi-squared test; *p* = .006). Conversely in rural subjects, *Prevotella spp*. were predominant, particularly in control subjects (15/35 rural subjects with >30% *Prevotella spp*. compared to 9/70 urban subjects, Chi-squared test; *p* = .001).

Analyzing the species-level taxonomic abundances using generalized linear models yielded 26 taxa which differed significantly between rural and urban control subjects, and 16 taxa which differed significantly between control and diarrheal subjects (Figure 2D, Supplementary Tables 2& 3). A direct comparison was also made between diarrheal subjects testing positive and negative for *C. difficile* toxin, yielding 18 taxa which differed significantly (Supplementary Table 4).

### Antimicrobial resistance is more prevalent in urban areas

**Figure 3.**
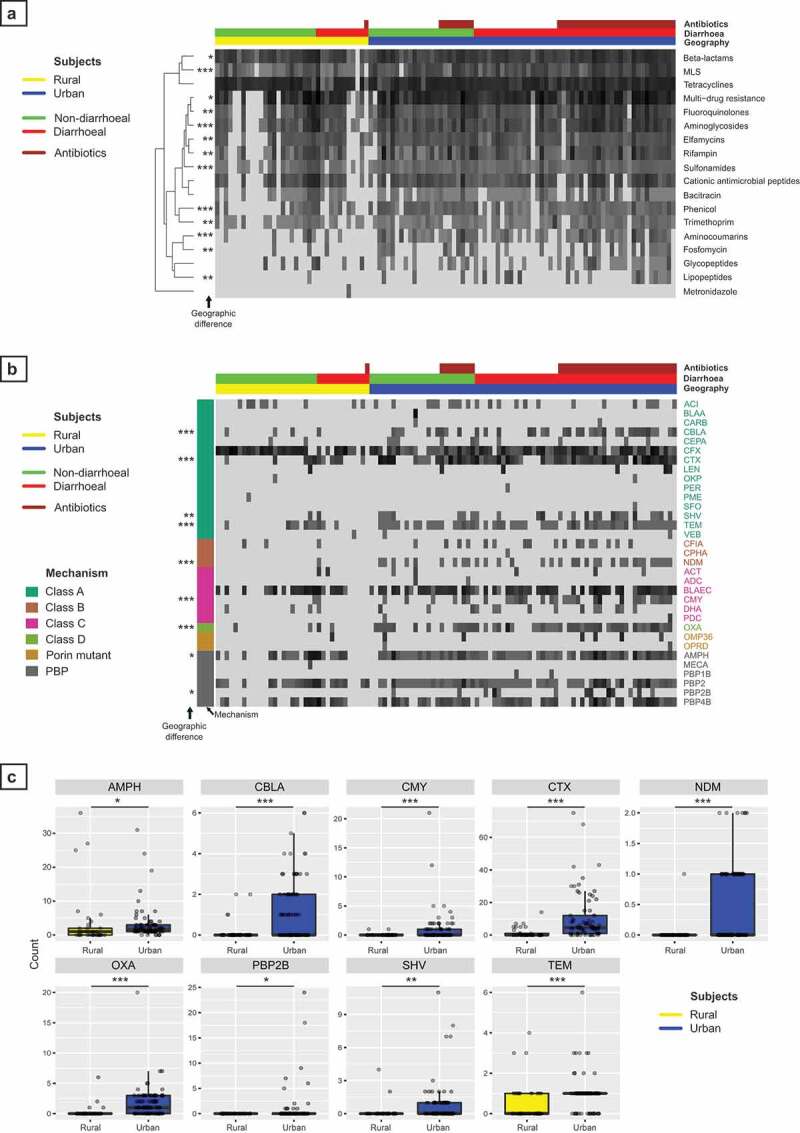
Analysis of antimicrobial resistance gene carriage by gut microbiota. (A) Heatmap of antimicrobial resistance (AMR) gene abundance aggregated by antibiotic class. Individual columns show subjects grouped by geography (rural – yellow vs. urban – blue), diarrheal status (non-diarrheal – green vs. diarrheal – red) and antibiotic exposure (brown). Row order represents hierarchical clustering of resistance gene count data using a Euclidean distance matrix. MLS = Macrolides, Lincosamides, and Streptogramins. (B) Heatmap of antimicrobial resistance gene cluster abundance for Beta-lactam antibiotics. Columns represent individual subjects, grouped as above. Individual gene cluster codes are shown in rows corresponding to MegaRes database entries. Beta-lactam resistance mechanisms for each gene cluster are indicated to the left of the heatmap; Ambler class A to D, Porin mutant or PBP (Penicillin Binding Protein). (C) Comparison of the Beta-lactam resistance gene counts which differed significantly between rural and urban subjects. All statistical comparisons between urban and rural subjects were made with the Mann–Whitney U test with FDR correction and results indicated in each panel. * *p* < .05, ** *p* < .01, *** *p* < .001.

Antimicrobial resistance gene profiles were compiled from the fecal metagenomes of all subjects in the study using ARIBA. Individual gene counts were aggregated by antibiotic class to identify broad trends between subjects according to geographic location and antibiotic exposure (Figure 3A). Genes conferring resistance to beta-lactam antibiotics, tetracyclines and macrolides, lincosamides and streptogramins (MLS) were identified in virtually all subjects. Average resistance gene counts aggregated by class were compared between subjects from rural and urban areas, regardless of diarrheal status or antibiotic exposure, indicating that counts for 13 of the 18 classes were significantly higher in urban subjects (Mann Whitney U test, FDR corrected, Figure 3A). Grouping subjects by geography, diarrheal status, and antibiotic exposure revealed a subset of rural subjects whose fecal metagenomes had resistance to the least number of different antibiotic classes, while some of the urban subjects were carrying antibiotic resistance genes to virtually every class of antibiotic (Figure 3A). This included resistance to glycopeptides (predominantly *vanA* genes) and two classes from the World Health Organization essential medicines reserve group; fosfomycin and lipopeptides (daptomycin). Compared with other antibiotic classes, metronidazole resistance was rare and only detected in a single subject.

Beta lactam antibiotics are widely used in clinical practice and resistance to broad spectrum beta lactam antibiotics, particularly carbapenems, is of significant public health concern. Individual beta lactam gene clusters derived from the MegaRes antibiotic database were analyzed in more detail by subject to identify differences in average gene counts between rural and urban subjects (Figure 3B), with those differing significantly shown in more detail in Figure 3C (Mann Whitney U test, FDR corrected). Resistance mechanisms included production of beta-lactamases (Ambler class A to D), alteration of penicillin-binding proteins (PBPs) and mutation of outer membrane porins in Gram-negative bacteria.

Of the gene clusters with increased counts in urban subjects, many encoded clinically relevant beta-lactamases, including extended spectrum beta-lactamases (CTX) and carbapenemases (NDM). Prevalence of key beta-lactamase genes was analyzed by comparing the number of subjects in which the gene cluster was detected in their metagenome. The CFX gene cluster, encoding an Ambler class A beta-lactamase, was the most prevalent cluster detected, identified in 94 of 105 subjects. The prevalence of several clinically relevant beta-lactam gene clusters was higher in urban subjects when compared to rural subjects, including CTX, NDM, and OXA (Supplementary Table 5). Gene clusters encoding the other clinically important carbapenemases, KPC, VIM, and IMP, were not detected in any of the subjects.

### Microbiota variations between groups are predicted to drive functional shifts in metabolic pathways

**Figure 4.**
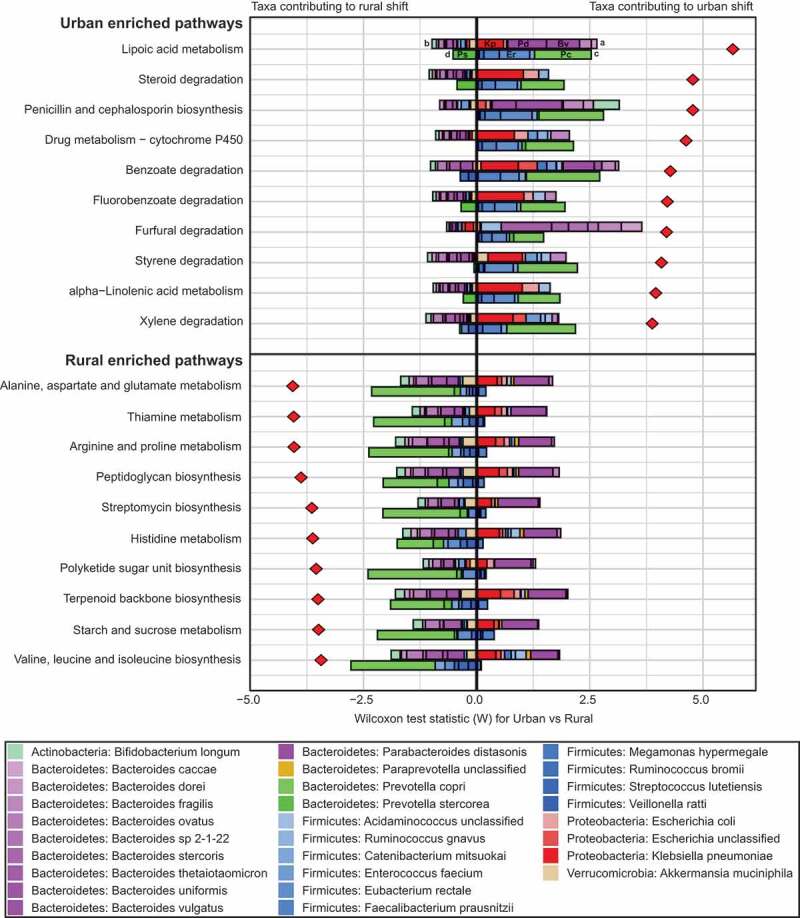
Taxonomic contributions to differentially enriched metabolic pathways. The top 10 pathways enriched in either urban or rural subjects are shown with the predicted contribution of individual taxa to the overall pathway variance (red diamonds). For each pathway, the top and bottom bars indicate urban- and rural-associated taxa, respectively, displaying the predicted contribution of each taxon to enrichment in either group; urban (positive) or rural (negative). For example, enrichment of Lipoic acid metabolism in urban subjects is associated with the positive contribution (A) of *Klebsiella pneumoniae* (Kp), *Parabacteroides distasonis* (Pd) and *Bacteroides vulgatus* (Bv), with only minor negative contributions from multiple other species (B). Rural-associated taxa contributing to enrichment in urban subjects (C), most likely because they encode the function sparsely, include *Prevotella copri* (Pc) and *Eubacterium rectale* (Er). *Prevotella stercorea* (Ps) is predicted to enrich this pathway in rural subjects (D), acting against the total observed shift.

Differentially abundant metabolic pathways between urban and rural subjects and their predicted taxonomic contributions were identified with FishTaco (Figure 4). A total of 28 pathways were enriched in urban subjects, with the majority (24/28) in the following categories; xenobiotics biodegradation and metabolism (16/28), lipid metabolism (6/28) and amino acid metabolism (2/28). Several *Bacteroides spp., Parabacteroides distasonis, Klebsiella pneumoniae*, and *E. coli* were identified as potential contributors to the enrichment of these pathways in urban subjects.

Of the 33 pathways enriched in rural subjects, 13/33 related to metabolism of amino acids, 4/33 to carbohydrate metabolism and 4/33 to metabolism of cofactors and vitamins. *Prevotella copri, Prevotella stercorea*, and several members of the *Firmicutes* phylum, including *Ruminococcus bromii, Eubacterium rectale*, and *Faecalibacterium prausnitzii*, were identified as potentially important contributors to the enrichment of these pathways in rural subjects, counterbalanced by the presence of *Parabacteroides distasonis* in urban subjects.

As the contribution of each taxa to the functional shifts had been inferred based on a comparison of taxonomic abundance to gene abundance across all samples, we sought further evidence based on the genomic content of related reference genomes to corroborate these findings. KEGG orthology copy number data for the top 10 urban and rural enriched metabolic pathways were obtained for 4 representative rural and urban genomes (Supplementary Tables 6 & 7). Several pathways relating to xenobiotics biodegradation and metabolism enriched in urban subjects were encoded at high copy number by the *Klebsiella pneumoniae* and *E. coli* reference genomes but were absent or encoded at low copy number by representative rural species, particularly *Prevotella copri*. For the rural enriched pathways, most were encoded at high copy number across all eight representative rural and urban species, consistent with the more balanced FishTaco profiles for these pathways. Although copy number by species for rural enriched pathways tended to be slightly higher for the urban representative species, their overall contribution may be offset by their relative abundance as a proportion of the total microbiota per subject.

Although no differences were identified in pathway enrichment between *C. difficile* positive and negative diarrheal subjects, 54 pathways were enriched in control non-diarrheal subjects when compared with diarrheal subjects. These included multiple pathway categories relating to amino acid metabolism (14/54), carbohydrate metabolism (10/54), cofactors and vitamins (8/54), and energy metabolism (6/54).

### Indian fecal viromes differ by geographic location

**Figure 5.**
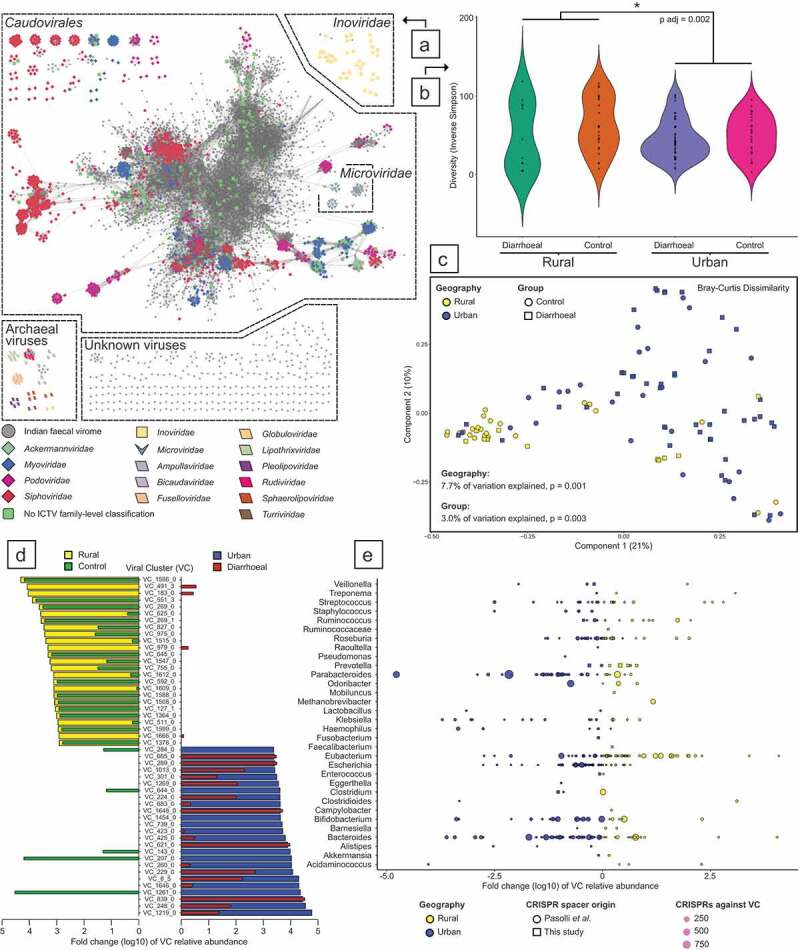
Contrasting fecal viromes by geographic location and diarrheal status. (A) Network visualization of viral clustering. Viral clusters (VCs) containing previously characterized viral sequences (viral RefSeq 85) are colored by International Committee on Taxonomy of Viruses (ICTV) family-level taxonomic assignments. While *Microviridae* VCs are connected to *Caudovirales* through shared protein clusters, these taxa are unrelated. (B) Inverse Simpson diversity comparisons of subjects by diarrheal status and geographic location. (C) Principal coordinate analysis of VC profiles based on Bray–Curtis Dissimilarity. (D) The fold change (log10) of the top 25 most abundant rural and urban VCs, with superimposition of the same VC’s association with either health or diarrheal status. (E) The fold change (log10) of all VCs relative abundance that is targeted by CRISPR spacers from identifiable bacterial genera. Each point represents a VC, with size representing the aggregate number of CRISPR spacers targeting individual viruses within a cluster.

A total of 8,746 non-redundant viral sequences were detected in the whole community metagenomic sequencing data for 105 Indian fecal samples. These viruses group into 1,344 Viral Clusters (VCs), which are concordant with viral genera.^22^ Network visualization of the shared protein clusters between VCs shows the majority of Indian fecal viruses identified are connected to previously described *Caudovirales* (Figure 5A). Several *Microviridae, Inoviridae*, and archaeal viruses of the *Rudiviridae* and *Bicaudaviridae* families, were also detected. Unknown viruses were observed which did not share protein clusters with previously characterized viruses.

As viruses were identified in whole community metagenomic data, and not specifically targeted using viral isolation and sequencing protocols, it is expected that rare viruses are poorly represented in the final Indian fecal virome. Therefore, for diversity comparisons between cohorts, the Inverse Simpson’s index was employed as it is less sensitive to rare taxa. No difference in viral diversity was observed between diarrheal and control subjects within specific residence locations. However, a difference in the Inverse Simpson’s index was detected between the rural and urban cohorts (rural mean 58.00 ± 37.53 versus urban mean 46.01 ± 25.36, p adj = 0.002; Figure 5B).

The unique composition of Indian fecal viromes was assessed through PCoA. The greatest variance is attributable to geographical residence, with 7.8% of the data explained by urban or rural location (F = 8.67, *p* = .001; Figure 5C). The interaction of geographical residence and the diarrheal status of subjects account for a further 2.1% of the observed viral differences (F = 2.36, *p* = .012). Amongst the urban and rural Indian cohorts that were suffering from diarrhea, the *C. difficile* status of individuals only accounted for an additional 0.6% of the PCoA variation (F = 0.63, *p* = .897). The impact of antibiotic usage with the geographical residence or diarrheal status of subject explains 1.0% and 1.4% of the calculated differences, respectively (F = 1.13, *p* = .315 and F = 1.64, *p* = .071, respectively). Additional recorded variables were tested for their effect on the Indian fecal virome. However, in combination, age, gender, BMI, and housing condition did not make a significant contribution to the variance explained, only accounting for 1.3% of the Indian fecal virome dissimilarities (F = 1.50, *p* = .09).

Specific VCs were strongly associated with distinct geographical locations and diarrheal status. The relative abundance differences observed for the 50 VCs that had the greatest fold change by geographical location demonstrates that specific VCs are also associated with controls (Figure 5D). Particular VCs associated with urban residing subjects were also clearly associated with diarrhea. Amongst individuals experiencing diarrhea, differences in the virome composition were noted between CDT positive and negative fecal samples (Supplementary Figure 3).

CRISPR spacers were used to link VCs to their potential bacterial hosts. The relative abundance of VCs and the number of CRISPR spacers against specific VCs demonstrate that urban subjects contain a greater abundance of phages targeting *Bacteroides, Parabacteroides, Bifidobacterium* and *Escherichia spp*., while there are trends toward more *Eubacterium* and *Prevotella*-infecting VCs amongst rural-residing individuals (Figure 5E). The enterotypes of Indian microbiomes (n = 105) are dominated by *Bacteroides* (n = 50), *Prevotella* (n = 34), and *Escherichia* (n = 21). When Indian fecal viromes are analyzed in the context of microbiome enterotypes, *Bacteroides-, Prevotella*-, and *Escherichia*-infecting phages are prevalent in the corresponding microbial enterotypes (Supplementary Figure 4a & B). Similarly, a trend toward more crAss-like phages predicted to infect *Prevotella* spp. are observed in rural samples (Supplementary Figure 4b; Kruskal–Wallis test, *p*-value 0.059).

### Virome-associated auxiliary metabolic functions

**Figure 6.**
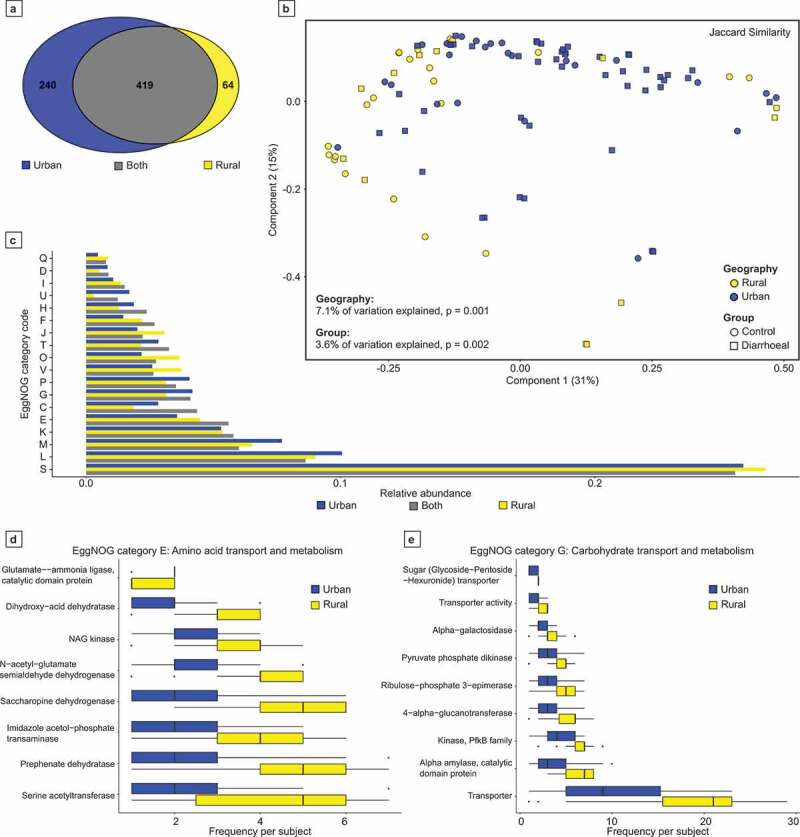
Examination of the auxiliary metabolic potential of human fecal viruses. (A) Shared proteins encoded by Viral Clusters (VCs) shared amongst 10 or more individuals within this study. (B) The VC-encoded metabolic functions were determined per individual virome, with the similarities between subjects visualized by principal coordinate analysis using the Jaccard index. (C) Relative abundance comparisons of the protein categorical-function predictions of VCs by residence. (D & E) The observed frequency of amino acid transport and metabolism functions, and carbohydrate transport and metabolism functional predictions encoded by individual virome VCs. Only statistically significant EggNOG functional predictions are displayed (Mann–Whitney U test with Bonferroni correction, p adj = 0.05).

While the Indian fecal virome composition analysis was conducted on VCs present in two or more individuals, all viral-associated auxiliary metabolic functions were assessed on VCs present in 10 or more individuals. These criteria were implemented in order to focus on the functions associated with the most abundant Indian fecal viruses. There were 723 VCs shared by 10 or more individuals. Of these VCs, the majority (419/723 VCs, 57.95%) are detectable amongst both rural and urban habiting individuals (Figure 6A). However, urban and rural-specific VCs were also observed (240 and 64 VCs, respectively).

The functions associated with the largest representative sequence of each VC were predicted. As expected for virome analyzes, the most abundant functional predictions corresponded to eggNOG category S: ‘Function unknown’ and category L: ‘Replication, recombination, and repair’ (Figure 6C). The presence/absence similarity between VC-encoded functions associated with an individual’s virome was compared using PCoA. The variation of the virome-associated auxiliary metabolic functions was better explained by geography than diarrheal status (7.1% versus 2.2%, *p* = .001 and 0.025, respectively; Figure 6B).

In order to assess the energy harvesting metabolic potential or urban and rural viral communities, eggNOG categories E and G (‘Amino acid transport and metabolism,’ and ‘Carbohydrate transport and metabolism,’ respectively) were investigated. The rurally abundant VCs encode at statistically higher frequency genes involved in amino acid and carbohydrate transport and metabolism (Figure 6D-E).

## Discussion

The composition of the gut microbiome in the context of health and to a much lesser extent, disease, in Indian populations is not well understood. This study is the first to utilize shotgun metagenomics sequencing to comprehensively characterize the gut bacteriome, resistome, and virome of rural and urban diarrheal and control populations without diarrhea living in two geographically and culturally distinct regions of Central India, Nagpur and Melghat. Although there are very limited data on the incidence and epidemiology of CDI in India as a whole, a handful of reports mainly conducted in hospitalized patients indicate detection rates in the range of 6–15.7%.^19–21^ In our fecal metagenome study in which we also sought to characterize the impact of *C. difficile*, we selected an enriched set of fecal DNA samples derived from diarrheal samples testing positive in diagnostic *C. difficile* immunoassays for whole-genome shotgun sequencing (WGS). As such, the *C. difficile* toxin positivity rates presented herein, may not reflect true prevalence rates in the selected study populations. Nevertheless, our results suggest that CDI is an emerging but as yet under-recognized healthcare-associated infection and is associated mainly with urbanization and antibiotic exposure. These findings highlight the need to enhance awareness of and testing of subjects with diarrhea for *C. difficile* in India, particularly in high-risk individuals with recent or ongoing antibiotic exposure or hospitalization.

The taxonomic profiles revealed geographically distinct gut microbiota signatures. As compared with the urban population of Nagpur district, the rural villagers of the Korku tribe in Melghat were observed to have a significantly higher abundance of *Prevotella spp*, particularly in the control subjects, and an underrepresentation of common members of urban-industrial gut microbiomes (e.g., *Bacteroides spp*.). *Prevotella* has been reported as the most prevalent genus associated with the healthy Indian population in previous microbiome studies^11,14,19,20^ and has also been observed as the dominant genus in Mongolian, Amerindian, and Malawian groups,^11^ indicating the occurrence of Enterotype 2 as proposed by Arumugam et al., 2011.^27^ *Prevotella* predominance may reflect the diet of the Korku tribe, which is rich in carbohydrates and dietary fibers. In contrast, Nagpur samples were associated with enterotype-1, which were driven by *Bacteroides* and may be again explained by this population’s dietary habits, which typically consists of rice, with some meat and fish. Interestingly, multivariate analysis revealed that geographic location actually accounted for most of the variation in gut microbial communities with diarrheal status, including *C. difficile* toxin positivity and antibiotics contributing to a lesser extent. Consistent with recent findings from a large-scale clinical microbiome study which surveyed over 7000 individuals across 14 districts within the Guangdong province in China,^28^ inter-individual differences in the composition of the gut microbiome could be overwhelmingly explained by an individual’s geographic location. Nevertheless, it is also now accepted that ethnicity strongly selects for specific taxa, although it is unclear what aspects of ethnicity, whether culturally related activities or genetics, underlie its observed association with the microbiota.^28,29^

The misuse and overuse of antibiotics in veterinary, agricultural, and clinical applications are rampant in India, fueling antimicrobial resistance. Inadequate public health infrastructure, poor sanitation, and infection control practices in the primary healthcare system increase demand for parallel markets and further contribute to the overuse of antibiotics. Antibiotic resistance is also being driven environmentally by untreated urban waste, sewage effluent from Indian hospitals,^30^ and pharmaceutical pollution of waterways.^31^ Indiscriminate use of beta-lactam antibiotics in both the community setting and hospitals has given rise to the presence of antibiotic-resistant *Enterobacteriaceae* in healthy human fecal samples in North India.^32^ Our fecal resistome data have corroborated recent shotgun metagenomics data indicating the widespread presence of AMR genes in virtually all subjects irrespective of geographic location and is consistent with that reported in Chinese, Hazda hunter-gatherer and resource-limited Latin American fecal microbiotas.^2,3,7^ However, although genes conferring resistance to beta-lactam antibiotics, tetracyclines, and macrolides, lincosamides and streptogramins (MLS) appeared to be common throughput Nagpur district and Melghat habitats, rural subjects from the Korku tribe generally reported lower exposure to antibiotics and thus displayed a lower abundance of other AMR genes compared with the urban Nagpur participants. In this latter group, those individuals with *C. difficile* infection on antibiotics were carrying AMR genes to virtually every antibiotic class.

The co-occurrence of pathogens and AMR genes for critically important antibiotics offers increased opportunities for unwanted horizontal gene transfer events.^30^ Perhaps of most concern, the Ambler class B metallo-beta-lactamase NDM, which was detected in only 1 of 35 rural subjects but was found in 32/70 urban subjects, and also supports clinical data detecting carbapenemase-producing pathogens from Mumbai,^33^ and another recent study showing that NDM-1 is also common in hospital effluent from Delhi.^34^ Our findings suggest that improving sanitation, health, and education as part of the UN Sustainable Development Goals as well as the consideration of new legislative measures for curtailing environmental pollution may be effective strategies for limiting the burden of AMR in India and globally.

Analysis of taxon-level shift contribution profiles in the Nagpurian population suggested that distinct bacteria such as *Bacteroides spp., Parabacteorides distasonis, Klebsiella pneumonia*, and *E. coli* may potentially possess xenobiotic, lipid, and amino acid metabolizing capabilities. In support of these observations, *Parabacteroides distasonis* has recently been shown to transform bile acids which have lipid-digestive and absorptive functions, and enhances the level of succinate in the gut. *Bacteroides spp*. are also dominant in amino acid metabolism in the large intestine.^35^ In addition, different species of *Klebsiella* appear to have substantial potential for the biodegradation of diverse pollutants, such as halogenated aromatic and nitroaromatic compounds.^36^ This result is in line with previous evidence, which suggests that individuals belonging to different geographies have microbiota with distinct xenobiotic metabolizing capacities.^37^ Our analysis of taxa associated shifts in metabolic function could also reflect diet and/or the higher exposure of these urban habitants to industrial/agricultural chemicals such as pesticides, fertilizers, antibiotics, and other pharmaceuticals.

Rural subjects tended to have a higher abundance of *Prevotella spp*. (and certain members of the Firmicutes phylum including *Roseuburia spp*. and *Eubacterium spp*.) and showed enrichment in pathways comprising amino acid and carbohydrate metabolism and metabolism of cofactors and vitamins. The FishTaco analysis indicated a potential association between these bacterial species and metabolic pathways. These observations are consistent with previous evidence indicating that *Prevotella spp*. show capacity to digest complex carbohydrates and display enzymatic potential to break down cellulose and xylan from foods.^38^ A specific strain, *Prevotella copri*, is one of the strongest driver species associated with branched chain amino acid biosynthesis in the gut and insulin resistance,^39^ and vitamin A and β-carotene from bananas and mangos can stimulate the growth of both *P. copri* and *P. stercorea*.^40^ Furthermore, the fecal metagenomes of the rural subjects were also enriched in genes associated with thiamine metabolism. It is feasible that thiamine deficiency, which is likely to be prevalent in the Korku, may be leading to a host driven compensatory increase in thiamine producing microbiota in the gut.

Ecological studies of macro-organisms consistently demonstrate the importance of predators within environments. Nonetheless, the majority of human microbiome studies only consider its bacterial fraction and do not concomitantly study this ecosystem’s predators, viruses. In this study, we identified and analyzed 8,746 viral sequences grouped into 1,344 putative genera termed Viral Clusters (VCs). Similar to previous studies of the human fecal virome, the vast majority of viruses detected are tailed phages of the order *Caudovirales* that infect bacteria (Figure 5A).

Phage predation has been proposed to modulate bacterial populations within ecosystems through various predator–prey interactions.^41,42^ The fecal virome diversity of Central India rural inhabitants was greater than their urban counterparts (Figure 5B). A similar observation is described by Rampelli *et al* (2017), whereby two hunter-gatherer communities also had a higher fecal viral diversity compared to two Western society cohorts.^43^

The changes in the relative abundance of VCs demonstrate specific viruses are strongly associated with urban and rural communities, and also with diarrheal status (Figure 5D). The identification of VCs’ host bacteria through CRISPR spacers is in agreement with the bacterial analysis of Indian fecal microbiomes. The relative abundance of viruses targeting *Bacteroides* and *Parabacteroides* is greater amongst urban residing individuals, while viruses targeting *Eubacterium* and *Prevotella* are more abundant amongst rural inhabitants (Figure 5E).

The abundance of unique proteins associated with VC representative sequences demonstrates the majority of functions are shared between urban and rural viruses (Figure 6A), with geography best explaining the observed differences (Figure 6B). The most abundant functional annotations associated with Indian fecal viromes correspond to ‘function unknown’ and ‘replication, recombination, and repair’ (Figure 6C). However, recent studies have highlighted the auxiliary metabolic potential of phages. Oceanic virome studies have demonstrated phages enhance the fitness of infected bacteria through augmenting their photosynthetic capability and energy production.^41,44^ Therefore, we investigated the energy harvesting potential encoded by human gut viruses. Specific pathways for amino acid and carbohydrate transport and metabolism are more abundant in rural VCs (Figure 6D & E). The increased abundance in rural associated VCs may be attributed to a narrower repertoire of encoded functions.

There were several limitations to this study. Co-morbidity data were unknown and we were unable to capture BMIs for all participants (see Supplementary metadata). Detailed dietary information was not available using a standard FFQ approach. Further, the control population comprised mainly hospitalized patients without diarrhea and thus do not represent healthy controls. It was also not possible to achieve identical sampling strategies across both rural and urban populations, particularly in view of lack of hospital facilities in Melghat. Due to lack of diagnostic facilities, we were unable to determine the etiological cause of acute diarrhea or in the case of *C. difficile* positive samples, undertake further strain characterization studies. Finally, due to limitations related to specimen collection and preparation, we were unable to assess other components of the microbiome, including RNA viruses and intestinal parasites.

## Conclusions

Here we report the most comprehensive study to date that has simultaneously examined the enteric bacteriome, DNA virome, and antibiotic resistome in divergent populations in Central India, a region of the world that has been grossly understudied. Together, these data suggest that not all rural traditional societies display a healthy gut microbiota as exemplified by a lack of significant difference in bacterial diversity between our rural and urban cohorts and the presence of a core set of AMR genes. Our findings will help assess progress toward meeting the goals of global and national action plans to tackle AMR and the burden of infectious diarrhea in India, including CDI. These results may also be useful in laying the foundations for implementing culturally acceptable One Health-inspired interventions to improve health-care outcomes in this region of the world.

## Materials and methods

### Experimental design and aim of study

The main aim of this observational cohort study was to use shotgun metagenomics to characterize the gut bacteriome, DNA virome, and antibiotic resistome of two highly divergent populations in Central India; rural agriculturalists in Melghat and an urban population in Nagpur. We also sought to investigate comparative differences in microbiome profiles in subjects with and without diarrhea, including the impact of CDI.

### Human participants

#### Inclusion and exclusion criteria

During participant selection, inclusion criteria were (i) adults aged from 18 to 70 years who could provide written or thumb-print acknowledged informed consent, (ii) HIV, hepatitis B or C negative, and (iii) not pregnant or breast-feeding.

For the diarrheal group, a presumptive diagnosis of infective diarrhea was defined as 3 or more loose stools in a 24-hour period accompanied by other gastrointestinal symptoms such as nausea, vomiting, abdominal cramps, tenesmus, bloody stools, or fever (oral temperature ≥38°C). All subjects in the *C. difficile*-infected group had diarrhea and a positive stool *C. difficile* (enzyme immunoassay) for toxin.

The exclusion criteria for this group were (i) any individual with a known noninfectious cause of diarrhea such as inflammatory bowel disease, (ii) those unable to provide a stool sample, (iii) or if the sample is formed stool. For the non-diarrheal control group, the exclusion criteria were (i) presence of acute diarrhea at the time of or within 2 weeks of recruitment or (ii) those unable to provide a stool sample. It was acknowledged that such individuals could be recruited from the in- or outpatient population and could have been exposed to antibiotics in the recent past (within 3 months of recruitment), although ideally not at the time of recruitment.

Immunosuppression was defined as those with cancer, were receiving chemotherapy or on prednisolone (>5 mg/d), immunomodulators (azathioprine, methotrexate, calcineurin inhibitor) or biologics.

Potential participants from Nagpur were identified with the assistance of project fellows at the Central India Institute of Medical Sciences (CIIMS) who approached all consecutive cases of diarrhea presenting to CIIMS as either an in-or outpatient. Similarly, all non-diarrheal cases were recruited to this study via the assistance of inpatient or outpatient clinical teams who closely liaised with the project fellows at CIIMS. All rural participants who provided stool samples in this study were directly recruited by community village health-care workers trained by MAHAN Trust, which is a non-governmental organization providing medical expertise to the disparate tribal population of the Melghat region in their own homes.

### Human geography – Nagpur

Nagpur is the third largest city of the Indian state of Maharashtra and the 13^th^ largest city by population (2.5 M) in India. It is located at the exact center of the Indian peninsula (zero milestone) and enjoys a tropical savannah climate where temperatures can reach in excess of 48°C in the summer months. Hinduism is the main religion followed closely by Buddhism and Islam, with smaller contributions from Christianity, Jainism, and Sikhism.

Nagpur is an emerging metropolis attracting significant commercial inward investment and is a major education hub in Central India. It is also home to the Central Indian Institute of Medical Sciences (CIIMS). Nagpur was declared open defecation free in January 2018 and is one of the cleanest and most livable cities in India, as a leader in healthcare, green spaces, and public transportation. The majority of households have good drinking water and sanitation facilities, and use clean fuel for cooking.

### Human geography – Melghat

Melghat Tiger Reserve, with its diverse flora and fauna, is located in Amaravati district of Maharashtra and is home to approximately 250,000 members of the Korku tribe spread across two talukas, Dharni and Chikaldhara and 300 villages, and extends across 4,000 square km. By road, it is approximately 250 km from Nagpur.

All rural Melghat subjects within the Melghat Tiger Reserve of Maharashtra identify as members of the Korku Scheduled Tribe and practice Hinduism mixed with ancestral worship. The Korku are an Adivasi ethnic group, speak Korku dialect, and are primarily an agriculturalist community of low socioeconomic status, high rates of illiteracy and malnutrition and possess poor access to medical and educational facilities. They live in small huts typically made of mud, grass, and bamboo frames which lack an electricity or running water supply or proper sanitation systems and possess unique and distinct cultural knowledge, beliefs, and customs.

### Metadata collection (Metagenome study)

Site-specific project coordinators were assigned to review health records form each participant. Basic demographic details including age, gender, geographic location, hospitalization exposure, antibiotic usage during and before (within 3 months) of study recruitment, and *C. difficile* (GDH positive, toxin-positive) detection rates were recorded for urban and rural diarrheal and control participants.

In addition, BMI, immunosuppression status, and environmental details: type and location of home dwelling, number in family, drinking water supply, hygiene practices, and number and type of domestic animals were also recorded for all participants. A description of the dietary information for the sampled cohorts is presented in the Supplementary methods.

### Fecal sample collection and storage

All specimens were anonymized and assigned a study code number linked to participant demographic details. Human fecal samples were collected from urban participants with and without diarrhea that were either in- or outpatients from the Central Indian Institute of Medical Sciences (CIIMS), Nagpur or from other hospitals within a 20 km radius of CIIMS. Similarly, fecal samples were also collected from participants with and without diarrhea in Melghat with the assistance of research fellows based at the Mahatma Gandhi Tribal Hospital, which hosts a CIIMS satellite laboratory and other neighboring hospitals within Melghat. Suitable recruits were identified by the research fellows who interacted daily with village health-care workers to facilitate participant recruitment and sample collection. Up to two samples (3–5 g each) were collected in UV sterilized dry plastic containers at the time of recruitment from each participant and placed in a cool box. As per the standard operating procedures, all stool specimens were stored at 4°C immediately after collection to avoid enzymatic degradation prior to the detection of toxigenic *C. difficile* and genomic DNA extraction which were performed within 24 hours of sample collection.

### Detection of Clostridioides difficile GDH antigen and free toxin in diarrheal stool samples

All diarrheal samples in the metagenome study (58/105) were tested for *Clostridioides difficile* infection (detection of glutamate dehydrogenase antigen and toxins A/B) using the C. DIFF QUIK CHEK COMPLETE-enzyme immunoassay (QCC; TechLab, Blacksburg, VA, USA) in accordance with the manufacturers’ instructions, including the use of appropriate controls as specified in the package insert. Briefly, ~25 ml of stool sample was added to a tube containing the diluent and conjugate and the mixture was transferred to the device sample well. After incubation for 15 min at room temperature, the wash buffer followed by the substrate was added to the reaction window. The results were read after 10 min. The GDH antigen and/or toxins were reported as positive if a clear visible band was seen on the antigen and toxin side of the device display window, respectively, confirming the presence of toxigenic *C. difficile* as per manufacturer guidelines.

### Fecal DNA extraction

DNA was extracted from 1 to 1.5 g of feces and homogenized in lysis buffer (Tris HCl, EDTA, NaCl, and SDS). The content was centrifuged at 7,000 *x g* for 10 min. The supernatant was then transferred to a 1.5 mL tube containing a mixture of Isopropanol and Sodium acetate (5 M) and incubated at −20°C for 30 min. Following removal of the supernatant the pellet was dried for about an hour. The pellet was suspended in 1X Tris EDTA buffer (pH 8) and incubated at 65°C for 15 min. An approximate equal volume (0.5–0.7 ml) of Phenol: Chloroform-Isoamyl alcohol (24:1) was added, mixed thoroughly and centrifuged for 10 min at 12,000 *x g*. The aqueous viscous supernatant was carefully transferred to a new 1.5 mL tube. An equal volume of Chloroform-Isoamyl alcohol (1:1) was added, followed by centrifugation for 10 min at 12,000 *x g*. The supernatant was mixed with 0.6x volume of Isopropanol to aid precipitation. The precipitated nucleic acids were washed with 75% ethanol, dried and re-suspended in 50 μL of TE buffer.

### Whole-genome shotgun (WGS) sequencing

Sequencing was carried out by Source Biosciences (Nottingham, U.K.). High-quality genomic DNA was quantified using Qubit Broad Range (Invitrogen, U.K.) and prepared for Illumina paired end sequencing following the TruSeq DNA Nano manufacturers protocol (Rev D, June 2015) (Illumina Inc, San Diego, U.S.A.). The DNA was sequenced using a standard HiSeq 4000 150bp PE flowcell. Raw data have been submitted to the European Nucleotide Archive under the accession number https://www.ncbi.nlm.nih.gov/bioproject/PRJNA564397

### Generation of taxonomic, resistome, and functional profiles from metagenomic shotgun data

Raw Fastq files (average 13,410,735 reads per sample) were assessed for quality using skewer,^45^ trimming adaptor reads and regions of quality below a phred of 30. The filtered reads (average 10,635,653 reads per sample) were then assessed for taxonomic assignments using Metaphlan2^46^ and for the presence of antimicrobial resistance genes using ARIBA^47^ with the MegaRes database.^48^

Functional analysis was performed using MOCAT2 (v2.1.3).^49^ Briefly, trimmed and filtered reads were assembled into contigs with SOAPaligner (v2.21). These contigs are initially corrected for indels and chimeric reads using BWA (v0.7.5a-r16) and screened against the human hg19 reference to filter out reads which originated from the host using USEARCH (v5/v6). Genes were predicted using Prodigal (v2.60). Single copy marker genes are extracted using fetchMG (v1.0) and clustered using CD-HIT (v4.6). The gene catalogs were annotated using DIAMOND (v0.7.9.58) against multiple functional databases including eggNOG^50^ and KEGG.^51^ The abundance of genes annotated to specific KEGG orthologs (KO) was determined using the insert mm dist among unique norm setting in MOCAT2, normalizing by read length and sequencing depth and allowing for multiple mappers.

### Analysis of taxonomic contributions to functional shifts

Functional shifts between groups and predicted taxonomic contributions were calculated using the FishTaco package,^52^ taking the species-level taxonomic table produced by Metaphlan2 and the normalized KO abundance table from MOCAT2 as inputs. Only 49 taxa which exceeded a minimum proportional abundance of greater than 0.1 in any single sample were included in the final model. Enriched pathways were identified using the Wilcoxon rank-sum test at FDR corrected *p* < .05. Taxonomic contributions were predicted by *de novo* inference in FishTaco, inferring genomic content through a permutation-based approach and performing a total of 50 permutations per differentially abundant pathway.

For comparison of gene copy number for enriched metabolic pathways, KO gene copy numbers for 8 gut-associated annotated reference genomes were obtained from the Integrated Microbial Genomes and Microbiomes (IMG) database^53^ as follows: *Prevotella stercorea* DSM 18206 (IMG: 2513237318), *Prevotella copri* CB7 DSM 18205 (IMG: 2562617166), *Eubacterium rectale* DSM 17629 (IMG: 650377936), *Ruminococcus bromii* L2-63 (IMG: 650377966), *Escherichia coli* UM147 (IMG: 2728369554), *Klebsiella pneumoniae* YH43 (IMG: 2687453226), *Bacteroides vulgatus* mpk (IMG: 2687453192), *Parabacteroides distasonis* 2b7A (IMG: 2660238380). KO gene copy numbers associated with each enriched metabolic pathway were aggregated to yield overall pathway gene counts.

### Detecting viruses in whole community metagenomic shotgun data

Sequencing reads were processed using Trimmomatic (version 0.36),^54^ to remove Illumina adaptors and prune sequences where the Phred score dropped below 30 across a 4bp sliding window. All surviving reads less than 70bp were discarded. Fastq reads were assessed pre- and post-processing using fastqc.^55^ Both the paired and unpaired, forward and reverse reads, from samples were assembled individually using metaSPAdes (version 3.11.1).^56^ Only contigs greater than 1,000 bp were examined further.

Two approaches were employed to find viruses within whole community metagenomic assemblies. A standard reference-based similarity search was performed to detect sequence relatedness to known viruses, while a reference-independent approach was undertaken by searching for sequences which encode a high density of viral proteins. For the reference-based search, nucleotide sequences were queried locally using BLAST (version 2.6.0+)^57^ against the viral RefSeq database (version 89; E-value 1E-10),^58^ the complete Reference Viral Database (C-RVDB version 14.0; E-value 1E-05),^59^ and 249 crAss-like phages previously described as the human gut’s most abundant viruses (E-value 1E-05).^60^

For the reference-independent approach, proteins for all contigs were predicted using Prodigal (version 2.6.3)^61^ with the ‘meta’ option enabled for small contigs and Shine-Dalgarno training bypassed. Proteins were subsequently queried against the prokaryote Viral Orthologous Groups database (pVOGs)^62^ using HMMER (version 3.1b2),^63^ with a minimum score requirement of 15. Putative reference-independent discovered viruses needed to fulfill three basic requirements: (i) ≥1.5kb, (ii) encode two distinct proteins with similarity to two unique pVOGs, and (iii) encode ≥2 pVOGs per 10kb-equivalent genome length. Additional stringent dynamic filtering was applied to contigs based on their actual genome length. For contigs <5kb, it was required that there were at least ≥5 distinct pVOG hits; contigs ≥5kb and <10kb, ≥6 pVOG hits; contigs ≥10kb and <20kb, ≥7 pVOG hits; contigs ≥20kb and <40kb, ≥8 pVOG hits; contigs ≥40kb and <60kb, 9 pVOG hits; and contigs ≥60kb, 10 pVOG hits.

All putative viral contigs detected using the reference-dependent and -independent methods were pooled and made non-redundant as follows: following a BLASTn all-v-all, the larger of two contigs were retained when the blast identity and coverage between two sequences exceeded 90%. Subsequently, any putative viruses encoding a ribosomal protein (BLASTp, E-value 1E-10) was removed from further analysis. This was performed for stringency despite recent research showing specific viruses can encode ribosomal proteins.^64^ In addition, any contig encoding a protein with similarity to all available Pfam sequences (version 32.0) of plasmid replication proteins PF01051, PF01446, PF01719, PF04796, PF05732, and PF06970, were removed (HMMER, score 15).

Viral contigs were grouped into Viral Clusters (VCs) using vContact2 (version 0.9.8),^65^ implemented through the CyVerse Discovery Environment. Protein clusters were identified amongst VCs using default settings (Diamond, E-value 0.0001), and with the inclusion of known viruses (Bacterial and Archaeal Viral RefSeq 85, with ICTV and NCBI taxonomy). Following vContact2, only viral clusters that contain viral sequences from two or more of the study’s complete cohort (n = 105) were analyzed further. This was designed to remove singleton and spurious viral sequences that may be transiently associated with diet, but are not abundant or stable components of the fecal microbiome. The final Indian fecal virome was visualized as a network through Cytoscape (version 3.7.1),^66^ with viral sequences as nodes and shared protein clusters as edges. The edge distance between connected viruses is calculated by Cytoscape as their ‘interaction.’

### Discerning differences in virome diversity and abundance

Quality filtered reads, both paired and unpaired, were mapped onto the final Indian fecal virome using bowtie2 in ‘end-to-end’ mode (version 2.3.4.1).^67^ The read alignment outputs were converted to sorted bam files through samtools (version 1.7).^68^ The abundance and breadth of coverage of reads mapping to each contig were determined using the bedtools coverage function (version 2.26.0).^69^ Subsequently, in order to determine if a viral sequence was indeed present in a fecal virome, a breadth of coverage filtering was applied. This was designed to remove viruses where potentially 100 s of reads could map onto a single conserved region. Therefore, for viral sequences ≤5kb, 75% of the genome needed to be covered by aligned reads; sequences >5kb and ≤50kb, 50% of the genome needed to be covered; and >50kb, 25% of the genome needed to be covered.

In addition to 105 fecal metagenomes, two negative control samples (water) were sequenced. While these samples contributed no contigs to the final Indian fecal virome, the breadth of coverage of sequencing reads from these samples was used to remove potential contaminant sequences. Any viral sequence, from any sample, which ‘passed’ the breadth of coverage filtering using reads derived from either water sample was removed from further analysis.

Any viral sequence from a fecal microbiome sample which failed the breadth of coverage filtering was recorded as zero reads, while if the filtering step was passed, the observed number of reads aligned was used to populate the read count matrix. Due to differences in sequencing depth between samples, the read count matrix was normalized per sample using the DESeq2 ratio of means method.^70^ The reads aligned to individual viral sequences were aggregated by their vContact2 determined VCs. DESeq2 was subsequently used to calculate the VC changes between cohorts. The normalized VC read count matrix was used to determine the diversity and statistical differences observed between Indian fecal microbiome cohorts (see ‘Statistical Analyzes’ below).

### Determining phage-host pairs and viral encoded functions

CRISPR spacers from bacterial contig assemblies were predicted using PILER-CR (version 1.06).^71^ Putative CRISPR spacer predictions <20 bp and >100 bp were discarded. The CRISPR spacers were queried locally using BLASTn against all individual viral sequences which formed the Indian fecal virome VCs. Due to the use of short nucleotide sequences, only CRISPR spacers with an E-value ≤0.001 and ≤1 mismatch were considered as significant. In order to determine the taxonomy of the original assembled bacterial contigs, or the pre-assembled contigs from the Pasolli *et al*. (2019) study,^72^ contig kmer MinHash sketches were queried against JGI taxonomy server using the BBMap sendsketch function (version 38.44).^73^ The bacterial enterotypes of Indian microbiomes were calculated using the Jensen–Shannon divergence (JSD) to cluster the samples, followed by partitioning around medoids (PAM) to cluster the abundance profiles.^27^

The functions associated with Indian fecal viruses were determined using eggNOG-mapper v1 (online submission portal) using the eggNOG 4.5.1 database.^50^ For each VC, the largest viral sequence was chosen as a representative of that VC. In order to avoid the confounding effect of viral abundance fluctuations within the fecal microbiome, the relative abundance of VCs observed at the specific sampling time-point was not taken into consideration. Only the overall presence-absence and abundance of viral-encoded functions were considered. The similarity between virome-encoded functions, with respect to presence-absence, was assessed through PCoA using the Jaccard index. The abundance of specific metabolic genes was compared between cohorts, with statistical difference determined by the Mann–Whitney U test with Bonferroni correction using the ‘ggpubr’ compare means function in R.

### Statistical analyzes and graphic generation

All statistical analyzes were conducted in R (64-bit, version 3.6.0; Foundation for Statistical Computing, Vienna). The package ‘vegan’ was used for measures of taxonomic diversity including alpha diversity (Inverse Simpson Index) and beta diversity (Principal Coordinates Analysis with Bray Curtis Dissimilarity and Jaccard Similarity). Differences in alpha diversity between study groups were assessed by ANOVA with Tukey’s honest significance test. The contribution of categorical variables to beta diversity was tested for using the Adonis function (PERMANOVA) in vegan. Comparisons of proportional carriage of key taxa and resistance genes between groups were assessed using the Chi-squared test. Generalized linear models assuming a negative binomial distribution were used to identify differentially abundant taxa between study groups as implemented in the R package ‘mare.’ Hierarchical clustering of resistance gene abundances and heatmap generation was performed with the package ‘heatmap3ʹ using log-transformed Euclidean distance for distance matrix construction from count data. For comparison of resistance gene and metabolic pathways counts between groups, the Mann–Whitney U test was used. All *p* values obtained from testing with multiple comparisons were corrected for false discovery rate (FDR, Benjamini-Hochberg). The fold changes observed in the relative abundances of VCs across geographical and diarrheal status cohorts were calculated using the ‘gtools’ package in R. Using the same package, the fold changes were converted to log ratios (base 10). All graphical images were generated using ‘ggplot2ʹ.

## Supplementary Material

Supplemental Material

## Data Availability

Metagenomic sequencing datasets generated and analyzed during the current study are available in the European Nucleotide Archive under accession number: [https://www.ncbi.nlm.nih.gov/bioproject/PRJNA564397] All sequencing reads that map to the human reference genome have been removed from the sequencing files.
