# Supplementary material for: Metagenomics reveals impact of geography and acute diarrheal disease on the Central Indian human gut microbiome
Source: Gut Microbes. 2020 May 27;12(1):1752605. doi: 10.1080/19490976.2020.1752605 (PMC7781581; doi:10.1080/19490976.2020.1752605)
Supplement: Supplemental Material [file KGMI_A_1752605_SM4574.docx]

**
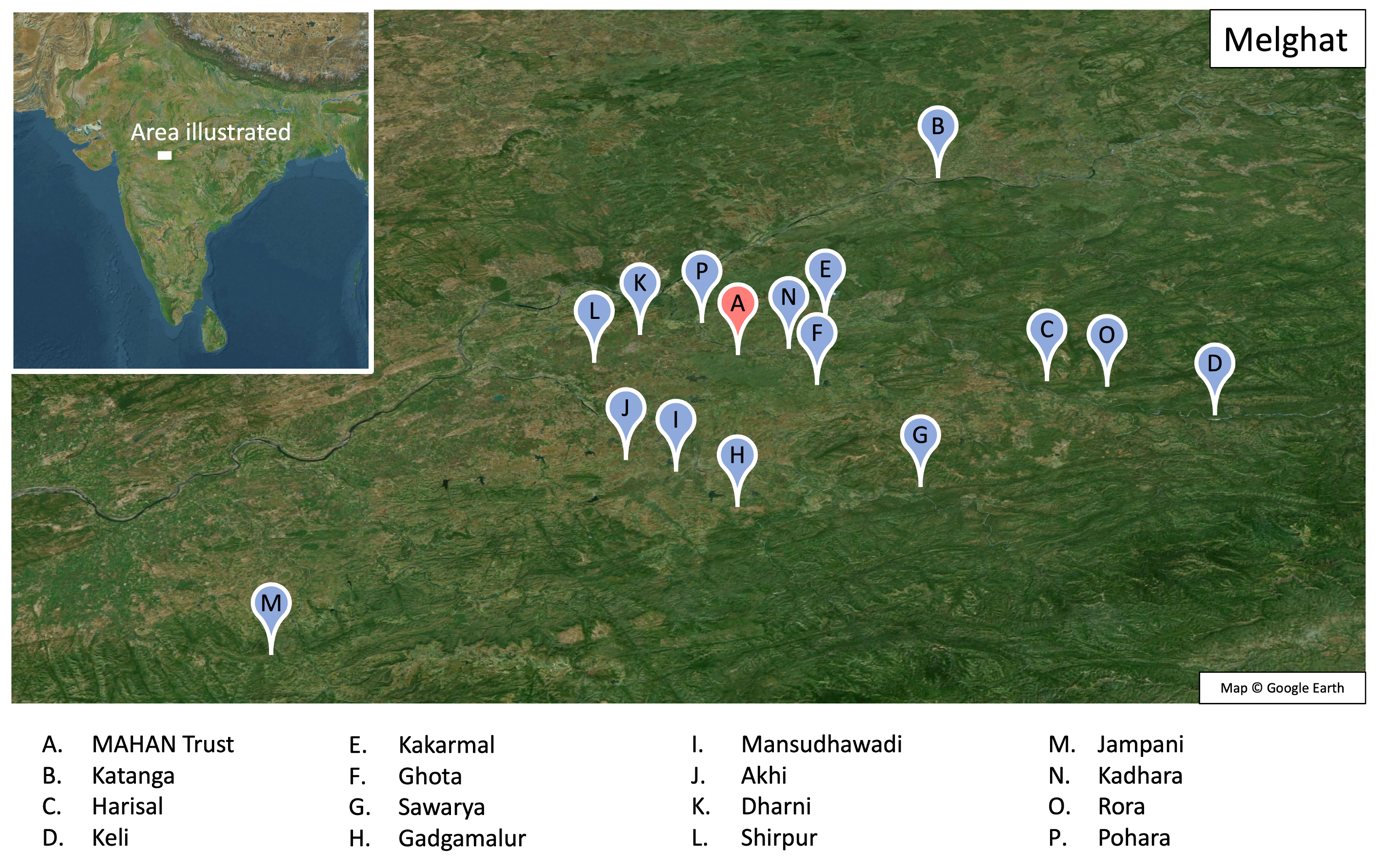
**

**SUPPLEMENTARY FIGURE 1.** Mapped locations of Melghat villages participating in the study

**
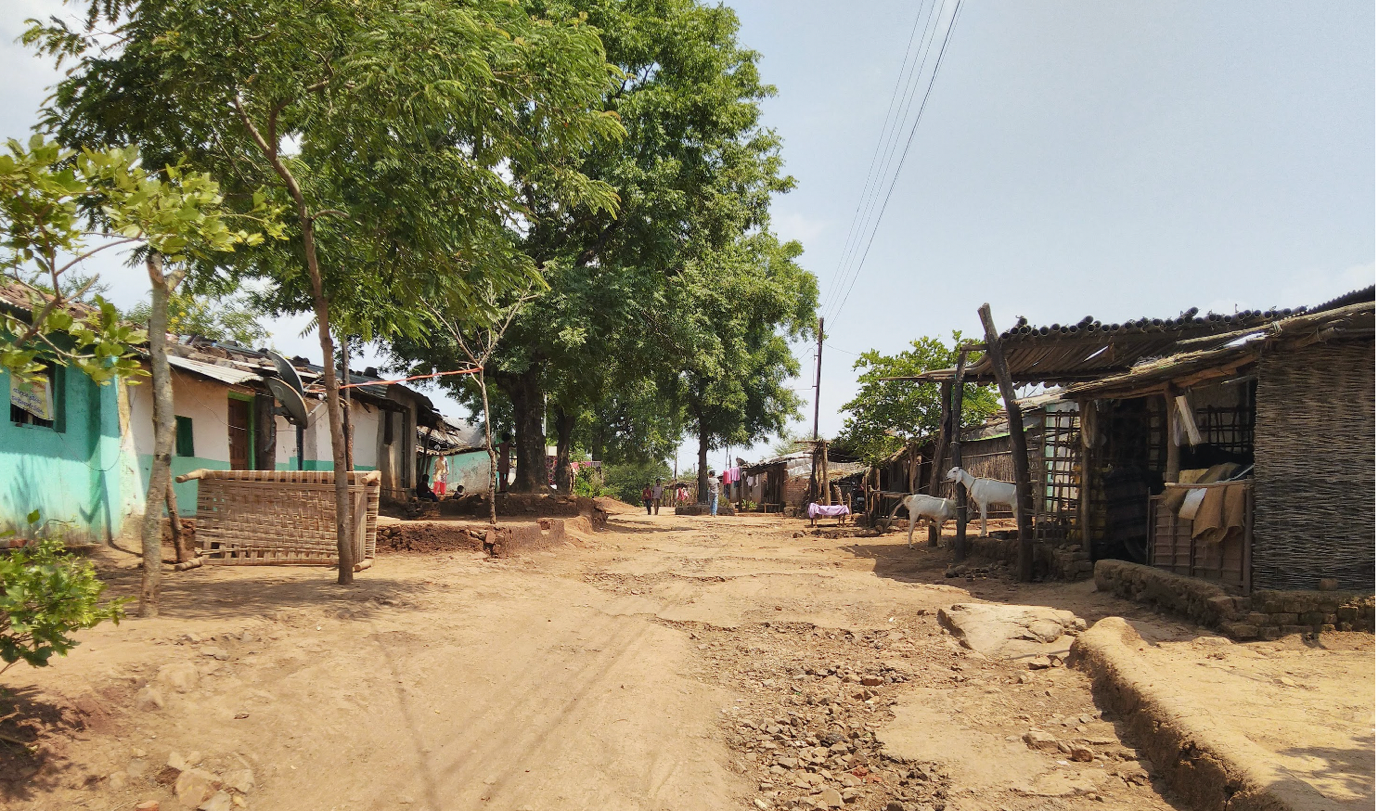
**

**SUPPLEMENTARY FIGURE 2.** Traditional Melghat tribal village


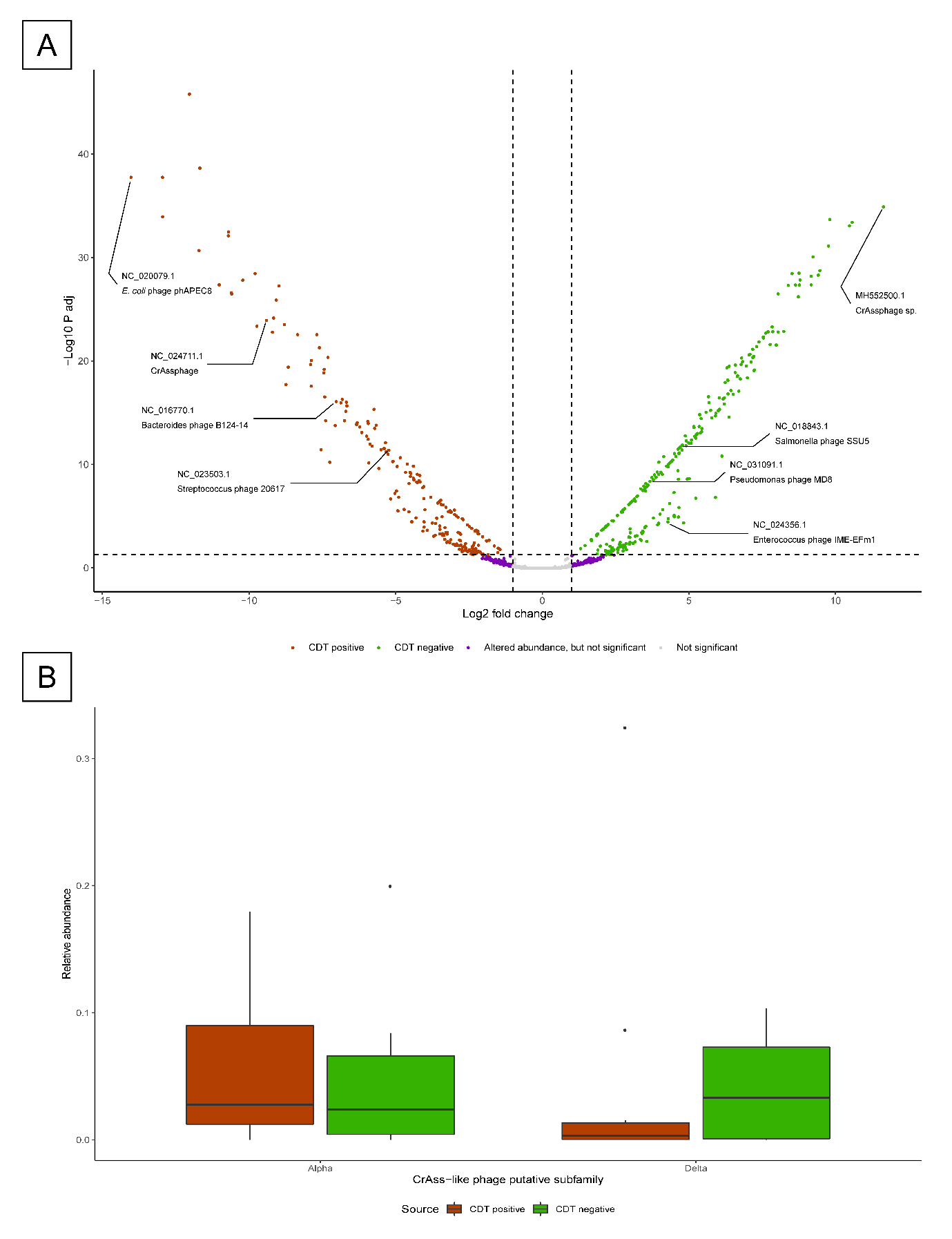


**SUPPLEMENTARY FIGURE 3.** Volcano plot of the fold change versus statistical significance of Viral Clusters (VCs). The top BLAST hit, and accession number, for several VCs are highlighted. **(B)** Examination of the relative abundance of putative crAss-like phage subfamilies alpha and delta, predicted to infect *Bacteroides* and *Prevotella*, respectively. No statistical differences were observed between crAss-like phage abundances by CDT status or geographical residence (Kruskal-Wallis test; CDT status: p-values 0.6 and 0.47, respectively; Geography: p-values 0.76 and 0.059, respectively).


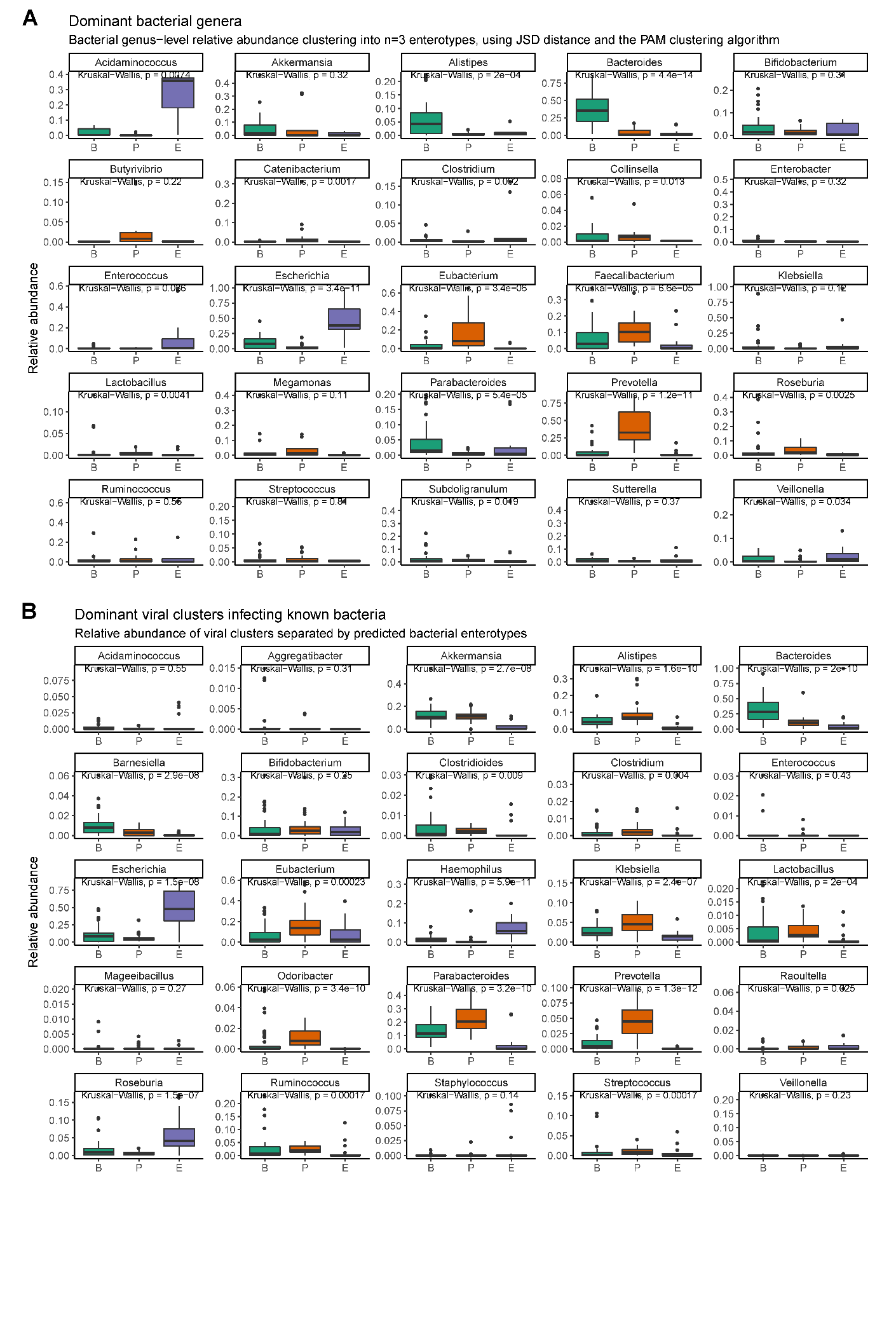


**SUPPLEMENTARY FIGURE 4.** Relative abundance of the top 25 most dominant (A) bacteria, and (B) viral clusters. Bacterial enterotypes (n=3) were dominated by *Bacteroides* (B), *Prevotella* (P), and *Escherichia* (E). Abundant viruses in faecal samples conforming to the B, P, and E enterotypes similarly infect *Bacteroides*, *Prevotella*, and *Escherichia*, respectively. However, for some lower abundant bacterial taxa (e.g. *Parabacteroides*), a corresponding abundance of its infecting viruses by enterotype was not observed.

**
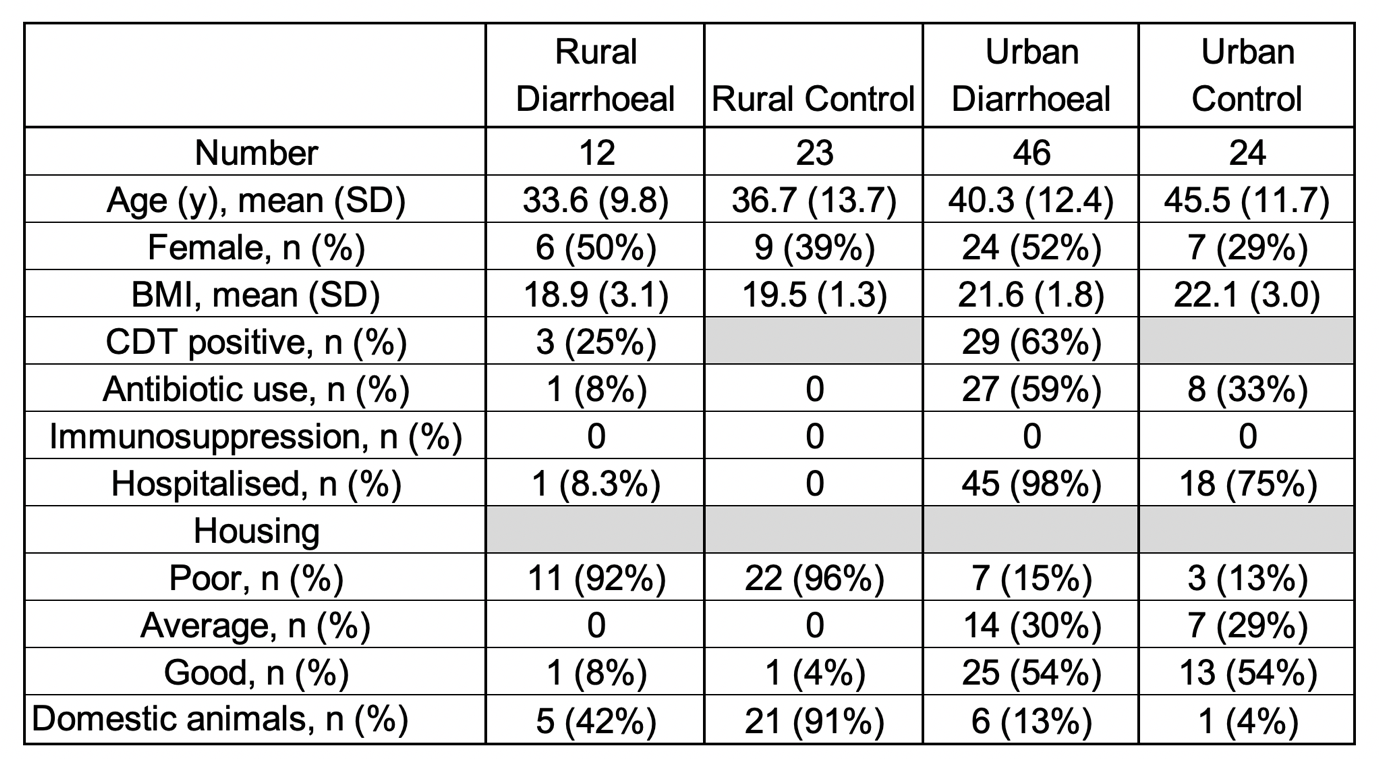
**

**SUPPLEMENTARY TABLE 1.** Baseline Cohort Characteristics for Metagenome Cohort

| **Taxa** | **Fold Change**  **Urban vs Rural** | **FDR p.corr** |
| --- | --- | --- |
| *Roseburia intestinalis* | 0.02 | 1.9x10^-7^ |
| *Bacteroides intestinalis* | 29.26 | 1.7x10^-5^ |
| *Megamonas funiformis* | 28.58 | 0.0001 |
| *Roseburia hominis* | 0.07 | 0.0007 |
| *Ruminococcus bromii* | 0.06 | 0.0014 |
| *Parabacteroides unclassified* | 11.50 | 0.0034 |
| *Lactobacillus ruminis* | 0.28 | 0.0083 |
| *Parabacteroides distasonis* | 9.23 | 0.0084 |
| *Catenibacterium mitsuokai* | 0.11 | 0.0125 |
| *Bacteroides thetaiotaomicron* | 8.42 | 0.0125 |
| *Veillonella parvula* | 8.94 | 0.0186 |
| *Enterobacter cloacae* | 8.28 | 0.0192 |
| *Subdoligranulum unclassified* | 3.98 | 0.0192 |
| *Prevotella stercorea* | 0.38 | 0.0225 |
| *Acidaminococcus intestini* | 0.12 | 0.0225 |
| *Megamonas hypermegale* | 9.28 | 0.0242 |
| *Haemophilus parainfluenzae* | 0.13 | 0.0252 |
| *Alistipes unclassified* | 0.15 | 0.0252 |
| *Escherichia unclassified* | 5.43 | 0.0342 |
| *Veillonella dispar* | 6.15 | 0.0422 |
| *Bacteroides vulgatus* | 2.49 | 0.0422 |
| *Alistipes finegoldii* | 6.01 | 0.0422 |
| *Paraprevotella clara* | 6.04 | 0.0422 |
| *Megamonas unclassified* | 7.32 | 0.0422 |
| *Bacteroides uniformis* | 5.31 | 0.0422 |
| *Enterococcus faecium* | 7.45 | 0.0422 |

**SUPPLEMENTARY TABLE 2.** Bacterial taxa enriched in Urban vs Rural subjects

| **Taxa** | **Fold Change**  **Diarrhoeal vs Non-diarrhoeal** | **FDR p.corr** |
| --- | --- | --- |
| *Eubacterium siraeum* | 0.04 | 1.81x10^-8^ |
| *Mitsuokella multacida* | 0.05 | 1.44x10^-7^ |
| *Barnesiella intestinihominis* | 0.09 | 0.0002 |
| *Collinsella aerofaciens* | 0.54 | 0.0017 |
| *Bacteroides ovatus* | 5.35 | 0.0050 |
| *Prevotella copri* | 0.35 | 0.0050 |
| *Bacteroides dorei* | 6.45 | 0.0069 |
| *Bacteroides uniformis* | 4.39 | 0.0132 |
| *Enterococcus faecium* | 5.48 | 0.0132 |
| *Ruminococcus bromii* | 0.20 | 0.0132 |
| *Bifidobacterium angulatum* | 0.23 | 0.0205 |
| *Bacteroidales bacterium ph8* | 0.22 | 0.0223 |
| *Subdoligranulum unclassified* | 0.40 | 0.0246 |
| *Clostridium nexile* | 4.64 | 0.0275 |
| *Roseburia intestinalis* | 4.16 | 0.0417 |
| *Bacteroides xylanisolvens* | 3.62 | 0.0448 |

**SUPPLEMENTARY TABLE 3.** Bacterial taxa enriched in Diarrhoeal vs Non-diarrhoeal subjects

| **Taxa** | **Fold Change**  **CDT Positive vs Negative** | **FDR p.corr** |
| --- | --- | --- |
| *Coprobacillus unclassified* | 35.23 | 1.72x10^-7^ |
| *Bacteroides ovatus* | 17.28 | 6.08x10^-6^ |
| *Lachnospiraceae bacterium* 2-1-58FAA | 22.90 | 1.42x10^-5^ |
| *Megamonas unclassified* | 0.05 | 0.0006 |
| *Catenibacterium mitsuokai* | 0.07 | 0.0009 |
| *Bacteroides fragilis* | 3.59 | 0.0017 |
| *Eubacterium eligens* | 0.09 | 0.0019 |
| *Enterococcus faecium* | 0.10 | 0.0046 |
| *Eubacterium rectale* | 0.32 | 0.0046 |
| *Barnesiella intestinihominis* | 0.11 | 0.0049 |
| *Bifidobacterium adolescentis* | 0.10 | 0.0060 |
| *Bifidobacterium breve* | 8.40 | 0.0113 |
| *Ruminococcus gnavus* | 7.71 | 0.0141 |
| *Alistipes indistinctus* | 8.35 | 0.0149 |
| *Bacteroides eggerthii* | 7.58 | 0.0190 |
| *Parabacteroides distasonis* | 5.85 | 0.0296 |
| *Dialister succinatiphilus* | 0.14 | 0.0335 |
| *Bacteroides intestinalis* | 5.95 | 0.0493 |

**SUPPLEMENTARY TABLE 4.** Bacterial taxa enriched in *C. difficile* toxin (CDT) positive subjects

| **Gene cluster** | **Rural** | | **Urban** | | **P value**  **(Chi-squared)** |
| --- | --- | --- | --- | --- | --- |
|  | **Proportion**  **(n=35)** | **Percentage**  **%** | **Proportion**  **(n=70)** | **Percentage**  **%** |  |
| **Ambler**  **Class A** |  |  |  |  |  |
| CTX | 11/35 | 31.4 | 58/70 | 82.9 | 5.3 x 10^-7^ |
| KPC | 0/35 | 0 | 0/70 | 0 | - |
| TEM | 10/35 | 28.6 | 53/70 | 75.7 | 9.1 x 10^-6^ |
| SHV | 2/35 | 5.7 | 25/70 | 35.7 | 0.002 |
|  |  |  |  |  |  |
| **Ambler**  **Class B** |  |  |  |  |  |
| IMP | 0/35 | 0 | 0/70 | 0 | - |
| NDM | 1/35 | 2.9 | 32/70 | 45.7 | 2.3 x 10^-5^ |
| VIM | 0/35 | 0 | 0/70 | 0 | - |
|  |  |  |  |  |  |
| **Ambler**  **Class C** |  |  |  |  |  |
| BLAEC/AmpC | 19/35 | 54.3 | 53/70 | 75.7 | 0.045 |
|  |  |  |  |  |  |
| **Ambler**  **Class D** |  |  |  |  |  |
| OXA | 4/35 | 11.4 | 48/70 | 68.6 | 1.1 x 10^-7^ |

**SUPPLEMENTARY TABLE 5.** Proportions of subjects with carriage detected of key beta-lactamase gene clusters

| **Pathway** | **Rural Associated Taxa** | | | | **Urban Associated Taxa** | | | | **Total Rural** | **Total Urban** |
| --- | --- | --- | --- | --- | --- | --- | --- | --- | --- | --- |
|  | **Ps** | **Pc** | **Er** | **Rb** | **Ec** | **Kp** | **Bv** | **Pd** |  |  |
| alpha-Linolenic acid metabolism | 0 | 0 | 1 | 0 | 5 | 6 | 0 | 1 | 1 | 12 |
| Benzoate degradation | 1 | 2 | 5 | 3 | 6 | 33 | 3 | 4 | 11 | 46 |
| Drug metabolism - cytochrome P450 | 0 | 0 | 1 | 0 | 8 | 11 | 1 | 1 | 1 | 21 |
| Fluorobenzoate degradation | 0 | 0 | 0 | 0 | 2 | 8 | 0 | 0 | 0 | 10 |
| Furfural degradation | 0 | 0 | 0 | 0 | 0 | 0 | 0 | 0 | 0 | 0 |
| Lipoic acid metabolism | 2 | 0 | 0 | 0 | 3 | 3 | 2 | 3 | 2 | 11 |
| Penicillin and cephalosporin biosynthesis | 0 | 0 | 0 | 0 | 0 | 0 | 2 | 0 | 0 | 2 |
| Steroid degradation | 0 | 0 | 0 | 0 | 1 | 1 | 0 | 0 | 0 | 2 |
| Styrene degradation | 0 | 0 | 0 | 0 | 0 | 3 | 0 | 0 | 0 | 3 |
| Xylene degradation | 0 | 0 | 2 | 1 | 0 | 9 | 1 | 0 | 3 | 10 |

**SUPPLEMENTARY TABLE 6.** Total kegg orthology (KO) gene copy numbers per pathway enriched in urban subjects for representative taxa from each group.

Ps = *Prevotella stercorea*, Pc = *Prevotella copri,* Er = *Eubacterium rectale*, Rb = *Ruminococcus bromii,* Ec = *Escherichia coli,* Kp = *Klebsiella pneumoniae*, Bv = *Bacteroides vulgatus,* Pd = *Parabacteroides distasonis*.

| **Pathway** | **Rural Associated Taxa** | | | | **Urban Associated Taxa** | | | | **Total Rural** | **Total Urban** |
| --- | --- | --- | --- | --- | --- | --- | --- | --- | --- | --- |
|  | **Ps** | **Pc** | **Er** | **Rb** | **Ec** | **Kp** | **Bv** | **Pd** |  |  |
| Alanine, aspartate and glutamate metabolism | 19 | 29 | 23 | 19 | 27 | 41 | 29 | 36 | 90 | 133 |
| Arginine and proline metabolism | 11 | 22 | 24 | 13 | 41 | 67 | 24 | 35 | 70 | 167 |
| Histidine metabolism | 5 | 9 | 11 | 12 | 10 | 16 | 13 | 15 | 37 | 54 |
| Peptidoglycan biosynthesis | 18 | 18 | 17 | 17 | 23 | 26 | 15 | 20 | 70 | 84 |
| Polyketide sugar unit biosynthesis | 5 | 4 | 4 | 2 | 5 | 10 | 4 | 4 | 15 | 23 |
| Starch and sucrose metabolism | 18 | 34 | 36 | 20 | 36 | 49 | 31 | 49 | 108 | 165 |
| Streptomycin biosynthesis | 7 | 7 | 6 | 4 | 20 | 18 | 10 | 13 | 24 | 61 |
| Terpenoid backbone biosynthesis | 14 | 15 | 13 | 10 | 16 | 17 | 13 | 17 | 52 | 63 |
| Thiamine metabolism | 10 | 12 | 14 | 10 | 13 | 16 | 10 | 8 | 46 | 47 |
| Valine, leucine and isoleucine biosynthesis | 1 | 11 | 13 | 11 | 17 | 20 | 9 | 15 | 36 | 61 |

**SUPPLEMENTARY TABLE 7.** Total kegg orthology (KO) gene copy numbers per pathway enriched in rural subjects for representative taxa from each group.

Ps = *Prevotella stercorea*, Pc = *Prevotella copri,* Er = *Eubacterium rectale*, Rb = *Ruminococcus bromii*, Ec = *Escherichia coli,* Kp = *Klebsiella pneumoniae*, Bv = *Bacteroides vulgatus*, Pd = *Parabacteroides distasoni*

**SUPPLEMENTARY MATERIALS**

**Dietary Information for Sampled Cohorts**

As donor participants provided samples to CIIMS from geographically dispersed sites across Nagpur and Melghat, it was not possible to systematically administer customised and standardised food frequency questionnaires to each participant. However, it was possible to elicit the major constituents of the inpatient diets based on knowledge of the principal foods provided within the hospital sector. A typical oral dietary hospital regime consisted of a morning beverage (tea, coconut water, fruit juice or lemonade), a lunchtime choice of oatmeal, rice porridge (semi-solid preparation of rice and cumin seed with coriander or basil leaves), semolina, curd rice or dal khichdi (1:2 proportion of pulses and rice added to water with salt and tumeric), an evening meal of black tea with Sago Kheer (sweet pudding made with tapioca pearls or sabudana and milk), and a late dinner which replicated the lunch menu. Beyond the hospital environment, the typical Nagpurian diet is vegetarian predominant and consists of a diverse mix of fruits, vegetables, grains, non-saturated fats and proteins. In contrast, the dietary repertoire of the rural Korku tribal participants is considerably narrower and typically consists of locally available plant-based foods rich in carbohydrates and high in fibre but low in protein content such as jowar ki roti made from millet flour and water in combination with various types of vegetable chutneys containing garlic, salt and green chillies. They generally feed twice daily, rarely consuming milk or meat, and usually eat the leftover food from the previous day.
